# Profound Metabolic Acidosis due to Metformin Intoxication Requiring Dialysis

**DOI:** 10.1155/2021/9914982

**Published:** 2021-05-24

**Authors:** Klodia Hermez, Carla Dudash-Mion

**Affiliations:** ^1^Michigan State University College of Osteopathic Medicine, 965 Wilson Rd, East Lansing, MI 48824, USA; ^2^McLaren Greater Lansing, 401 W. Greenlawn Avenue, Lansing, MI 48910, USA; ^3^Ingham Nephrology and Hypertension, 405 W Greenlawn Ave # 230, Lansing, MI 48910, USA

## Abstract

Metformin-associated lactic acidosis (MALA) is a rare but life-threatening condition with often high mortality rates. Despite this, metformin continues to be one of the most commonly prescribed antihyperglycemic agents in the market. We present a unique case of a 61-year-old female with severe acidosis of pH = 6.72 and lactic acid of 26 mmol/L who presented obtunded after ingestion of an unknown amount of metformin. She was subsequently intubated, became hypotensive, and was initiated on vasopressors. She was swiftly started on a combination of intermittent hemodialysis (IHD) and bicarbonate therapy 7 hours after admission followed by continuous renal replacement therapy (CRRT) as she became more hemodynamically unstable. The patient's renal function improved, and she was discharged 7 days after admission with favorable sequelae. Dialysis is often reported in cases of severe MALA; however, it remains unclear how quickly dialysis should be initiated. This case aims to explore the benefits of quick initiation of extracorporeal measures in the forms of IHD and CRRT with concurrent bicarbonate supplementation. Furthermore, this case demonstrates the importance of clinical suspicion in metabolic acidosis in a patient on metformin therapy.

## 1. Introduction

Metformin (Glucophage) is a first-line antihyperglycemic agent that is frequently prescribed in the United States for type 2 diabetes mellitus [1]. The medication operates by promoting euglycemia and inhibiting gluconeogenesis in the liver, as well as increasing insulin sensitivity [1, 2]. Despite its relative safety and efficacy, toxic ingestions of metformin have been reported in the literature with often high morbidity and mortality [2–6]. Metformin-associated lactic acidosis (MALA) has been coined as the reason for renal damage as well as multisystem organ failure often leading to death in such cases [2]. This mechanism is thought to be due to impaired mitochondrial function [7]. Furthermore, the safety of metformin with chronic kidney disease has recently come into question, with mortality being higher in those with creatinine clearance of <45 ml/min/1.73 m^2^ [1]. While MALA has been reported in the literature, mainly causing severe renal failure and metabolic acidosis, the time between initiation of renal replacement therapy (RRT) and impact on outcomes is less clear. Although correcting acidosis is likely correlated with the correction of pH and stabilization of the patient, the time frame in which to start dialysis remains unclear. Additionally, bicarbonate supplementation and its effects on mortality in MALA are unclear. This case serves to demonstrate that quick initiation of appropriate extracorporeal measures to support the patient is of paramount importance in order to reduce mortality. The patient presented with suspected metformin overdose and was initiated on dialysis within 7 hours of presentation, which likely contributed to her favorable outcome. Mortality for this patient without such measures is predicted to have been as high as 80% [6].

## 2. Case Presentation

A 61-year-old female with type II diabetes mellitus presented to the emergency department 3 hours after ingestion of an unknown amount of medication, suspected to be metformin. The patient's pertinent medical history also includes hypertension, depression, and schizoaffective disorder. The patient was encephalopathic on presentation, and her husband stated that the patient was confused after waking up from a nap which alarmed him. She had previously tried to overdose on metformin 9 months prior. Her medications consisted of atorvastatin 20 mg p.o. daily, hydroxyzine pamoate 50 mg p.o. BID, Invega 3 mg p.o. QHS, Invega Sustenna 156 mg/mL IM monthly, Levemir 100 units/mL subcutaneous solution 10 units QAM, lisinopril 20 mg p.o. daily, and metformin 850 mg p.o. BID.

Physical exam showed her to be moderately distressed, moaning in bed. She had slurred speech but was able to follow simple commands. The cardiorespiratory system was unremarkable; the abdomen was soft and nontender. Blood pressure was 116/43 mmHg, heart rate was 88 beats/minute, and respiratory rate was 24 breaths/minute; she was afebrile, with oxygenation of 100% on 2 liters nasal cannula. Laboratory workup showed profound metabolic acidosis: arterial blood gas with pH of 6.728, pCO_2_ of 10.2 mmHg, and pO_2_ of 170 mmHg. Actual bicarbonate was <5 mmHg, anion gap was “unable to be calculated,” and lactic acid was 19.9 mmol/L. In addition to the above, her potassium was 6.3 mmol/L, and creatinine was 11.8 mg/dL (baseline is 0.87 mg/dL). The nephrology service was contacted and recommended initiation of a bicarbonate drip at 150 mEq in sterile water. She received 100 mEq of sodium bicarbonate 8.4% IV push in the ED. The patient's remaining lab values are detailed in Table 1. She underwent a CT chest/abdomen/pelvis without contrast which showed right lower lobe pneumonia and spiculated and nonobstructing renal calculus. CT of the head without contrast was negative for any acute intracranial abnormality. Acute toxicology screen was negative, and assay for the serum metformin level was not available at our facility.

**Figure 1:**
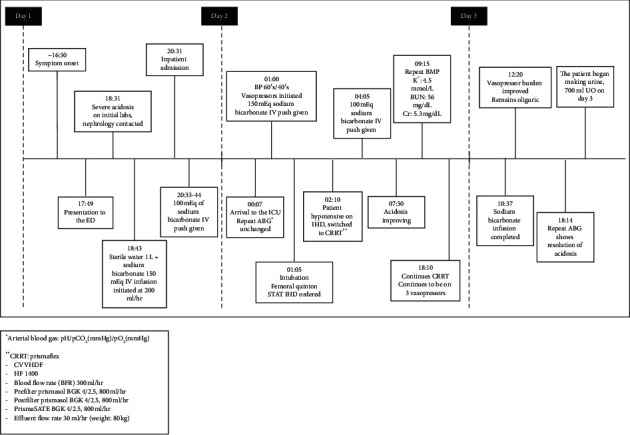
Timeline of clinical progression.

On arrival to the intensive care unit, she was hypotensive and only responsive to sternal rub. She was subsequently intubated, and vasopressors were initiated with norepinephrine and phenylephrine infusions. At this point, nephrology was contacted again and initiated hemodialysis, which was approximately 7 hours after presentation. The patient's repeat arterial blood gas showed pH of 6.74 with worsening lactic acidosis of 26 mmol/L. She was initiated on intermittent hemodialysis (IHD). The patient became hypotensive and bradycardic and was maximized on three vasopressors after 30 minutes. A decision was made by nephrology to switch to continuous renal replacement therapy (CRRT). The patient received 150 mEq of bicarbonate IV push at initiation of dialysis, 2 g calcium chloride, and 0.5 mg of atropine. Additional 100 mEq of bicarbonate IV push was given 3 hours after initiation of CRRT. A detailed timeline of the patient's presentation is demonstrated in Figure 1. Details of dialysis modalities and prescription are also provided in Figure 1.

The patient stabilized and tolerated CRRT well with normalization of her acidosis after two days and was successfully extubated on day 6. She underwent CRRT for four days after which she underwent one day of IHD. Her creatinine slowly improved with adequate urine output. She was discharged in a favorable condition on day 7 with instructions to follow up with nephrology and avoid metformin in the future at all costs.

## 3. Discussion

This case of MALA is unique in two aspects: pH of 6.72 on admission and swiftness with which RRT was initiated. Numerous cases exist describing MALA; however, data looking at RRT initiation remain scarce. A study conducted by Peters et al., looking at acute and chronic ingestions of metformin, was inconclusive in regard to the advantage of RRT in MALA [8]. One study conducted in 2020 discussed how early RRT is beneficial especially with pH < 7.0, lactic acid concentration >20 mmol/L, and failure of standard supportive measures [9, 10]. The role of RRT was reported to be crucial in removing metformin and correcting acidosis, with benefit seen in continuous removal at a lower effluent rate. Furthermore, a study conducted in Italy looking at 117 patients admitted for MALA-AKI with RRT showed high survival rate, nearly 80% [11]. Average time between entry to ICU and RRT was 3.2 hours, with concurrence that prolonged methods of dialysis such as CRRT were more beneficial. Moreover, our time of ICU admission to initiation of RRT was about 1 hour, significantly less than the average reported above, demonstrating a more aggressive approach.

**Figure 2:**
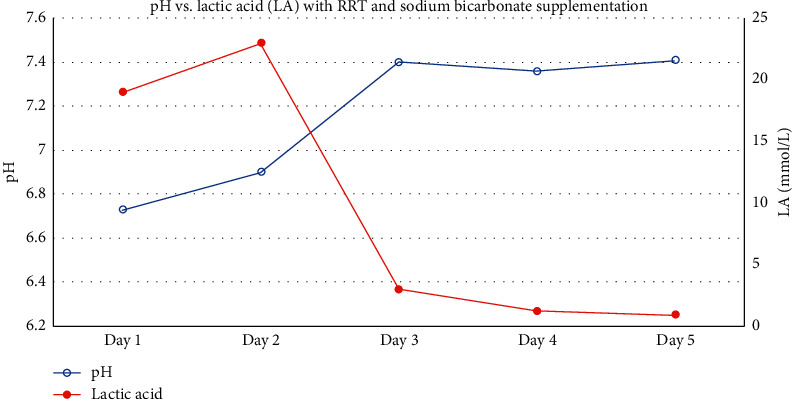
pH vs. lactic acid (LA) with RRT and sodium bicarbonate supplementation.

**Figure 3:**
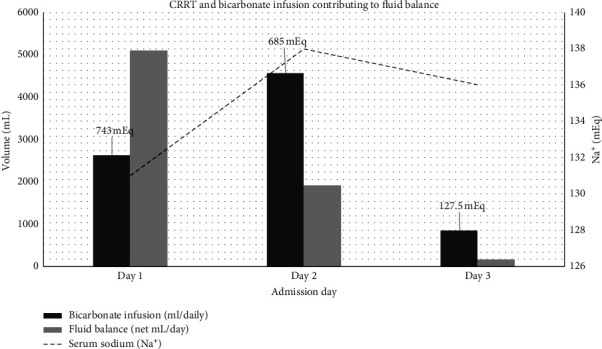
CRRT and bicarbonate infusion contributing to fluid balance.

In addition to the discussion above, metformin has a large volume of distribution, so early RRT may be beneficial before the substance distributes throughout the body and tissues [12]. As metformin metabolites cause a severe increase in lactic acid, mortality soars. In fact, after a lactic acid value of 20 mmol/dl, every increase in the value results in added 9% mortality [6]. IHD is seen as superior as it clears metabolites faster; however, it is used less in extreme cases due to hemodynamic compromise of severe MALA [13]. Furthermore, due to the multifactorial nature of severe lactic acidosis, it is unclear if the mortality trend is linked to RRT alone. In our particular case, initiation of IHD with concurrent bicarbonate replacement was unsuccessful as the patient could not tolerate more than 30 minutes before becoming hypotensive. CRRT appears to have been effective with continuous IV bicarbonate replacement as demonstrated in Figures 1 and 2. Additionally, CRRT appears to have been vital in preventing hypernatremia due to large amounts of sodium bicarbonate administered to correct acidosis. In our particular case, the trends of sodium bicarbonate supplementation vs. fluid balance and sodium levels are demonstrated in Figure 3. This theory is corroborated by Mariano et al. discussed above. Sodium bicarbonate supplementation with and independent of RRT was a significant treatment modality used in this case. In the first 72 hours, this patient received approximately 1,500 mEq of sodium bicarbonate in IV push and infusion combined. There does not exist sufficient evidence to show whether sodium bicarbonate supplementation is correlated with better outcomes in MALA [14]. Additionally, documentation of bicarbonate supplementation and mortality have been investigated in case reports in adolescents with MALA; however, the benefits have not been fully explored in adults [15].

This case took place at a community hospital with limited resources, so a measured metformin level was not available. While history was difficult to obtain from the patient due to her altered mentation, the previous suicide attempt with metformin coupled with her physical exam and lab findings prompted the clinicians to treat the patient as a metformin overdose. This demonstrates the importance of acting on clinical suspicion and initiating appropriate treatment. Currently, no predictive models exist for the treatment of MALA and when to initiate dialysis. Further detailed studies are needed to investigate the utility of RRT in metformin overdose, especially in regard to time from ingestion to clearance. As metformin continues to be widely prescribed, further education is necessary to better prepare clinicians and patients to combat this lethal syndrome.

## Figures and Tables

**Table 1:** Lab values.

Laboratory variables	Labs on admission	Labs on discharge	Normal values
PCO_2_ (mmHg)	10.2	37.7	37–43
HCO_3_ (mmol/L)	1.30	24.3	22–28
pH	6.728	7.426	7.37–7.44
WBC (×10^9^/L)	17.7	11.1	4–12
Hemoglobin (g/dl)	9.2	8.8	12.3–15.7
Platelets (×10^9^/L)	318	521	150–440
Sodium (mEq/L)	131	139	135–145
Potassium (mEq/L)	6.3	3.3	3.5–5.1
Chloride (mEq/L)	85	104	92–109
Blood sugar (mg/dl)	235	77	60–100
HbA1C (%)	9.1	N/A	3–5
Anion gap	44.7	12	8–12
Serum albumin	2.4	1.8	3.4–5.4
BUN (mg/dL)	99	23	8–25
Creatinine (mg/dL)	11.8	2.02	0.5–1.5
Lactic acid (mmol/L)	19.9	0.5	0.5–2.2
Salicylate level (mg/dL)	<2	N/A	<2
Troponin (ng/ml)	0.037	N/A	0–0.017
Calcium (mg/dl)	8.3	9.1	8–10.4
AST (units/L)	18	106	10–40
ALT (units/L)	22	257	7–56
Alkaline phosphatase (units/L)	78	289	25–115
Total protein (g/dL)	5.9	5.9	5.6–8.4
Magnesium (mg/dL)	2.9	2.4	1.6–2.8

## Data Availability

Data availability is restricted due to the patient's privacy. See attached figures and tables.

## References

[1] Rhee C. M., Kovesdy C. P., Kalantar-Zadeh K. (2017). Risks of metformin in type 2 diabetes and chronic kidney disease: lessons learned from taiwanese data. *Nephron*.

[2] Rena G., Hardie D. G., Pearson E. R. (2017). The mechanisms of action of metformin. *Diabetologia*.

[3] Kopec K. T., Kowalski M. J. (2013). Metformin-associated lactic acidosis (MALA): case files of the einstein medical center medical toxicology fellowship. *Journal of Medical Toxicology*.

[4] Dell’Aglio D. M., Perino L. J., Kazzi Z., Abramson J., Schwartz M. D., Morgan B. W. (2009). Acute metformin overdose: examining serum pH, lactate level, and metformin concentrations in survivors versus nonsurvivors: a systematic review of the literature. *Ann Emerg Med*.

[5] Taub E. S., Hoffman R. S., Manini A. F. (2019). Incidence and risk factors for hyperlactatemia in ED patients with acute metformin overdose. *The American Journal of Emergency Medicine*.

[6] Yeh H.-C., Ting I.-W., Tsai C.-W., Wu J.-Y., Kuo C.-C. (2017). Serum lactate level and mortality in metformin-associated lactic acidosis requiring renal replacement therapy: a systematic review of case reports and case series. *BMC Nephrology*.

[7] Orban J.-C., Fontaine E., Ichai C. (2012). Metformin overdose: time to move on. *Critical Care*.

[8] Peters N., Jay N., Barraud D. (2008). Metformin-associated lactic acidosis in an intensive care unit. *Critical Care*.

[9] Mariano F., Biancone L. (2020). Metformin, chronic nephropathy and lactic acidosis: a multi-faceted issue for the nephrologist. *Journal of Nephrology*.

[10] Calello D. P., Liu K. D., Wiegand T. J. (2015). Extracorporeal treatment for metformin poisoning. *Critical Care Medicine*.

[11] Mariano F., Pozzato M., Inguaggiato P. (2017). Metformin-associated lactic acidosis undergoing renal replacement therapy in intensive care units: a five-million population-based study in the north-west of Italy. *Blood Purification*.

[12] Liu S., Xu L., Ma J. (2018). High-volume continuous venovenous hemodiafiltration plus resin hemoperfusion improves severe metformin-associated toxicity. *Journal of Diabetes Investigation*.

[13] Chiew A. L., Wright D. F. B., Dobos N. M. (2018). ’Massive’ metformin overdose. *British Journal of Clinical Pharmacology*.

[14] Heaney D., Majid A., Junor B. (1997). Bicarbonate haemodialysis as a treatment of metformin overdose. *Nephrology Dialysis Transplantation*.

[15] Harvey B., Hickman C., Hinson G., Ralph T., Mayer A. (2005). Severe lactic acidosis complicating metformin overdose successfully treated with high-volume venovenous hemofiltration and aggressive alkalinization. *Pediatric Critical Care Medicine*.

